# 1688. Naturally-induced Serum Antibody Levels in Children to Pneumococcal Polysaccharide 15B that Correlate with Protection from Nasopharyngeal Colonization but Anti-serotype 15B Antibody has Low Functional Cross-reactivity with Serotype 15C.

**DOI:** 10.1093/ofid/ofad500.1521

**Published:** 2023-11-27

**Authors:** Ravinder Kaur, Eduardo Gonzalez, Minh Pham, Michael Pichichero

**Affiliations:** Rochester General Hospital Research Institute, Rochester, New York; Rochester General Hospital Research Institute, Rochester, New York; San Francisco State University, San Francisco, California; Rochester General Hospital Research Institute, Rochester, New York

## Abstract

**Background:**

Pneumococcal infections caused by non-vaccine serotypes, including serotype 15B and 15C, emerged after the introduction of pneumococcal conjugate vaccines (PCVs). Serotypes 15B and 15C have been added to new different pneumococcal-conjugate vaccines (PCV20 and V116, respectively). We sought to derive a serum anti-15B antibody level that would be a correlate of protection (COP) against acute otitis media (AOM) and nasopharyngeal colonization and assess functional cross-reactivity against serotype 15B and 15C in children following natural immunization.

**Methods:**

Method: IgG antibody to serotype15B polysaccharide was measured by ELISA in 402 sera from 6-36 month olds collected before, at the time of, and after pneumococcal culture-confirmed AOM and colonization caused by serotypes 15B and 15C. 153 serum samples from 6-36 month olds where no colonization with either 15B or 15C was observed were included as controls. A two-step method was used for the construction of COP models: a generalized estimating equation followed by logistic-regression. Opsonophagocytic (OPA) assays were used to assess functional cross-reactivity between serotypes 15B and 15C.

**Results:**

The COP for prevention of colonization was 1.41µg/ml for 15B and 0.63µg/ml for 15C with a predictive probability of 80%. Significantly higher anti-15B antibody levels were measured in children who had AOM caused by serotype 15B compared to control children and compared to children who had AOM caused by serotype 15C (p< 0.03). Consequently, a COP for serotypes 15B and 15C could not be calculated for AOM. ELISA-measured antibody levels did not correlate with OPA titers. Thirty percent of child samples, with varying moderate to high amounts of ELISA-measured antibody, showed no OPA titer against either serotype 15B or 15C. For the remaining samples, very low or no functional cross-reactivity between serotypes 15B and 15C was measured.

**Conclusion:**

Conclusions: A COP for prevention of colonization in young children based on naturally-induced antibody levels was derived for serotype 15B and 15C that differed. A COP for AOM could not be derived. ELISA-measured antibody levels correlated poorly with OPA titers and low functional cross-reactivity between serotypes 15B and 15C in child sera was observed.

**Disclosures:**

**All Authors**: No reported disclosures

